# Seasonal Changes in Anthropometry, Body Composition, and Physical Fitness and the Relationships with Sporting Success in Young Sub-Elite Judo Athletes: An Exploratory Study

**DOI:** 10.3390/ijerph17197169

**Published:** 2020-09-30

**Authors:** Olaf Prieske, Helmi Chaabene, Martijn Gäbler, Michael Herz, Norman Helm, Adrian Markov, Urs Granacher

**Affiliations:** 1 Division of Exercise and Movement, University of Applied Sciences for Sport and Management Potsdam, Am Luftschiffhafen 1, 14471 Potsdam, Germany; prieske@fhsmp.de; 2 Division of Training and Movement Sciences, Research Focus Cognitive Sciences, University of Potsdam, Am Neuen Palais 10, 14469 Potsdam, Germany; chaabanehelmi@hotmail.fr (H.C.); m.gabler@umcg.nl (M.G.); miherz@uni-potsdam.de (M.H.); norman.helm@osp-brandenburg.de (N.H.); adrian.markov@uni-potsdam.de (A.M.); 3 Department of Human Movement Sciences, University Medical Center Groningen, University of Groningen, Antonius Deusinglaan 1, 9713 AV Groningen, The Netherlands; 4 Olympic Testing and Training Center Brandenburg, Olympischer Weg 2, 14471 Potsdam, Germany

**Keywords:** combat sports, periodization, somatic variables, training load, training monitoring, young athletes

## Abstract

This exploratory study aimed to monitor long-term seasonal developments in measures of anthropometry, body composition, and physical fitness in young judo athletes, and to compute associations between these measures and sporting success. Forty-four young judoka (20 females, 24 males) volunteered to participate. Tests for the assessment of anthropometry (e.g., body height/mass), body-composition (e.g., lean body mass), muscle strength (isometric handgrip strength), vertical jumping (e.g., countermovement-jump (CMJ) height), and dynamic balance (Y-balance test) were conducted at the beginning and end of a 10-month training season. Additionally, sporting success at the end of the season was recorded for each athlete. Analyses revealed significant time × sex interaction effects for lean-body-mass, isometric handgrip strength, and CMJ height (0.7 ≤ *d* ≤ 1.6). Post-hoc analyses showed larger gains for all measures in young males (1.9 ≤ *d* ≤6.0) compared with females (*d* = 2.4) across the season. Additionally, significant increases in body height and mass as well as Y-balance test scores were found from pre-to-post-test (1.2 ≤ *d* ≤4.3), irrespective of sex. Further, non-significant small-to-moderate-sized correlations were identified between changes in anthropometry/body composition/physical fitness and sporting success (*p* > 0.05; −0.34 ≤ *ρ* ≤ 0.32). Regression analysis confirmed that no model significantly predicted sporting success. Ten months of judo training and/or growth/maturation contributed to significant changes in anthropometry, body composition, and physical fitness, particularly in young male judo athletes.

## 1. Introduction

Judo is one of the most popular grappling combat sports worldwide [1]. It was officially included as part of the summer Olympic Games in 1964 (Tokyo) for males and in 1992 (Barcelona) for females [2]. Judo’s activity profile requires the athlete to perform repetitive high-intensity efforts in standing and groundwork positions interspersed by periods of lower intensity and/or rest [3]. The maximal duration of the official judo match is 4 min for both sexes [4]. Despite the relatively short duration of a judo match, the physiological demands are remarkably high [5]. In a comprehensive review of the literature, Franchini et al. [3] indicated that judo affords high levels of muscle strength, power, and cardiorespiratory endurance to achieve success in competition, irrespective of sex. In this context, Drid et al. [6] compared fitness and anthropometric profiles between male elite (international medalists) and sub-elite (national medalists) judo athletes. The authors revealed that elite judoka were stronger and more powerful than their sub-elite peers. Additionally, the same authors reported higher muscle mass in the upper limb in elite compared with sub-elite athletes [6]. Thus, tracking fitness profiles and anthropometry on a regular basis over the course of a training season in judo athletes appears to be critical since these characteristics drive performance during competition [3,6,7]. In fact, these data are essential to help coaches evaluate their training on a daily basis by tailoring ongoing decision-making processes [8]. This becomes even more important in young judoka given that the main long-term goal of training with young athletes is to lay the foundation for higher training loads at the elite level [9].

Of note, a recently published cross-sectional study indicated that anthropometric characteristics (e.g., body mass, fat mass) and training among others significantly predicted performance in fitness tests such as standing long jump (*R*^2^ = 35%) or medicine ball push (*R*^2^ = 53%) in male judo athletes aged 10 to 16 years [10]. In terms of training, few experimental studies are available that have investigated the short-term (i.e., 8–13 weeks) effects of different periodization models using unspecific training modalities (e.g., strength training) in addition to specific training modalities (e.g., randori, technical/tactical training) on components of physical fitness and judo-specific performance in young judoka [11,12]. However, there is a void in the literature with regards to observational studies that describe the long-term development (e.g., across a training season) of regular judo training on measures of anthropometry, body composition, and components of physical fitness in young judoka and how these changes are associated with sporting success during competition (e.g., tournament ranking).

In addition, the lack of studies in female judoka represents an intriguing shortcoming in the scientific literature. In fact, most of the previous studies have predominantly focused on young male judo athletes [10,12]. Even more, no study has contrasted changes in anthropometry, body composition, and physical fitness between males and females over one training season [11,12]. Of note, girls differ from boys in terms of body composition (e.g., percentage body fat), metabolic performance (i.e., lower aerobic capacities), and hormonal status (e.g., lower testosterone level) [13,14]. Thus, it is not possible to directly translate the short-term effects of judo training in young male judoka to training-induced, long-term effects in young female judo athletes.

It is noteworthy that the long-term effects of judo training on anthropometry, body composition, and components of physical fitness have not been explored previously in the literature in male versus female young judoka (e.g., changes across an entire training season). In addition, it is unresolved if at all or how these measures are related to success in competition. Therefore, the main goal of this exploratory study was to assess changes in anthropometry (i.e., body mass, body height), body composition (i.e., lean body mass, and fat mass), and physical fitness (i.e., maximal strength, vertical jump performance, and dynamic balance) in young male and female elite judoka over the course of one training season (i.e., 10 months). The second aim of the study was to examine the relationship between the above-mentioned parameters and sporting success.

## 2. Materials and Methods

### 2.1. Design and Procedures

In this exploratory study, a single group repeated measures design was used to systematically monitor seasonal developments of anthropometry, body composition, and physical fitness in young judo athletes. Following the school year, the observation period lasted 10 months. It started from a first preparation period in September 2016 to a third competition period with peak performance in June 2017. Training data were regularly documented by the coaches over the entire training period in an online data management system. In addition, the results of the main competition at peak performance were registered. Young athletes were tested at the beginning (i.e., at the onset of preparation period) and the end (i.e., around main competition) of the season using a standardized test battery. Tests included the assessment of anthropometry, body composition, and components of physical fitness. The anthropometric measurements comprised the assessment of standing and sitting body height, body mass, and body-mass-index (BMI). Additionally, body composition was analyzed for lean body mass, relative fat mass, and lean upper and lower limb mass. Of note, judo is considered as a late specialization sport [15]. Accordingly, non-specific, generic fitness tests were used to lay a broad foundation of physical fitness in young judo athletes. More specifically, physical fitness testing comprised the measurement of maximal muscle strength (isometric handgrip strength), vertical jump performance (countermovement jump (CMJ) and drop-jump (DJ) height, DJ contact time, and DJ reactive strength index (RSI)), and dynamic balance (Y-balance test score). Isometric handgrip strength has been shown to be a good indicator of a subject’s general muscle strength [16]. Additionally, jump tests (e.g., CMJ) have been reported to be valid and reliable which could be easily used on the field to predict lower limb muscle power [17]. In terms of the Y-balance test, it has been demonstrated to provide reliable outcomes in youth [18]. Prior to testing, a standardized warm-up protocol (i.e., 15 min of running, jumping, change-of-direction drills, and dynamic stretching) was performed.

### 2.2. Participants

Twenty female (age: 14.1 ± 0.9 years) and 24 male (age: 14.1 ± 1.2 years) adolescent sub-elite judo athletes from an elite sport school in Germany volunteered to participate in this study. Participants’ maturity status was estimated using the maturity offset method. In brief, maturity status was determined according to the regression equations of Mirwald et al. [19]:

Boys’ maturity offset = −9.236 + (0.0002708 × leg length × sitting height) − (0.001663 × age × leg length) + (0.007216 × age × sitting height) + (0.02292 × weight by height ratio)

Girls’ maturity offset = −9.376 + (0.0001882 × leg length × sitting height) + (0.0022 × age × leg length) + (0.005841 × age × sitting height) − (0.002658 × age × weight) + (0.07693 × weight by height ratio)

Male athletes were classified as pre-peak height velocity (PHV) (*n* = 4), circa-PHV (*n* = 13), and post-PHV (*n* = 7). Further, female athletes were classified as circa-PHV (*n* = 2) and post-PHV (*n* = 18). Before the start of the study, written informed consent was obtained from the participants and their parents/guardians. All procedures were approved by the local ethical committee for the use of human participants (submission No. 5/2014). The study was conducted in accordance with the latest version of the Declaration of Helsinki.

### 2.3. Assessment of Anthropometry and Body Composition

In Judo, the regular monitoring of measures of anthropometry (e.g., body mass) and body composition (e.g., lean body mass) during training is essential for sporting success [20]. Therefore, we recorded measures of anthropometry (i.e., standing/sitting body height, body mass) and body composition (i.e., lean body mass, fat mass, upper/lower limb lean mass) using a stadiometer (seca, Hamburg, Germany) and a bioimpedance analysis system (InBody720 system, Biospace, Seoul, South Korea), respectively. The test of body composition can be classified as highly reliable with an intraclass correlation coefficient (ICC) of ≥0.98 for lean body mass and body fat [21]. Additionally, leg length (i.e., iliac height) was assessed at the beginning of the test sessions. Standardized testing procedures for anthropometry and body composition were always conducted in a fasted state between 7:00 and 10:00 a.m.

### 2.4. Assessment of Physical Fitness

#### 2.4.1. Maximal Strength

As a measure of maximal strength, participants’ isometric handgrip strength was assessed while sitting on a chair with a digital hand dynamometer (JAMAR^®^ Plus+, Sammons Preston, Bolingbrook, IL, USA) in the dominant hand and the respective elbow flexed at 90° [16]. High test–retest reliability (ICC of 0.99) was reported for the assessment of isometric handgrip strength using a digital hand dynamometer [22]. Hand dominance was defined according to the lateral preference inventory [23]. During testing, participants had to press the hand dynamometer as forcefully as possible for 3 s. Three test trials were performed and the best was used for further analysis.

#### 2.4.2. Vertical Jump Performance

Countermovement jump and DJ performances were determined as indicators of lower limb muscle power using an optoelectric device (Optojump, Microgate, Bolzano, Italy). High test–retest reliability with an ICC value of 0.99 was previously reported for measuring vertical jump height during a CMJ with an Optojump system [24]. In terms of CMJ, participants stood in an upright erect position, feet shoulder-width apart, and hands akimbo. Participants initiated the jump with a rapid countermovement by flexing the hip, knee, and ankle joints, which was immediately followed by an explosive hip, knee, and ankle extension. For the DJ, participants stood in an upright erect position on a 40 cm box, feet shoulder-width apart, and hands akimbo. Participants stepped off the box with their dominant leg, landed evenly on both feet on the ground, and jumped quickly off the ground with a double-leg vertical jump at maximal effort. Leg dominance was defined according to the lateral preference inventory [23]. All participants were instructed to jump as high as possible (CMJ, DJ), to land with extended legs, and to minimize ground contact time (DJ). Proper care was taken to ensure a uniform dropping technique for all participants (e.g., extended legs during flight time, jumping in the vertical direction) [25]. Jump height was calculated using the formula: jump height = 1/8 × g × t^2^, with g representing the acceleration due to gravity and t representing the flight time. [26] Three jump trials were conducted with a rest period of 30 s between trials and a 1 min rest between CMJ and DJ. Three trials were allowed per jump. The highest values for vertical jump height (CMJ and DJ) and reactive strength index (RSI = jump height by corresponding ground contact time during DJ) were used for further data analyses.

#### 2.4.3. Dynamic Balance

Dynamic balance was quantified utilizing the Y-balance test (Move2Perform, Evansville, IN, USA) [27]. Before testing, participants’ left and right leg lengths were assessed in supine lying position by measuring the distance from the anterior superior iliac spine to the most distal aspect of the medial malleolus. Additionally, participants performed three familiarization trials per reach direction on each foot. During testing, all trials were conducted barefoot. Participants were positioned in single-leg stance on a Y-balance test tool (Move2Perform, Evansville, IN, USA) while reaching as far as possible with the contralateral leg in three different reach directions (i.e., anterior, posteromedial, posterolateral). High test–retest reliability was reported for the Y-balance test in all three movement directions with ICC values ranging between 0.89 and 0.93 [28]. Validity was previously confirmed in young and older adults [29,30]. Participants always started reaching as far as possible with the left leg and three times per reach direction. Afterward, standing and reaching legs were changed. The examiner manually measured the distance from the scale of the tool. A composite score was calculated and used as a dependent variable for further data analysis [27]. The composite score was computed for the dominant and non-dominant limb using the following formula: composite score = [(maximum anterior reach distance + maximum posteromedial reach distance + maximum posterolateral reach distance)/(leg length × 3)] × 100.

### 2.5. Monitoring of Training and Competition Data

Team coaches recorded day-to-day training data (i.e., volume, type) for each training session on the group level over the entire season using online training logs. Training types were coded as strength training, endurance training, sprint training, flexibility training, technical training, tactical training, and randori. Further, the type of strength training was classified as heavy-resistance strength training, power training, or training of muscular endurance. Training volume was summarized as hours spent per week, and the type of strength training (heavy-resistance, power, muscular endurance) was expressed relative to total strength training volume.

To determine sporting success, each athlete was evaluated with regards to their performance during the main international tournament which was scheduled at the end of the competitive season. More precisely, the individual rank during the competition was evaluated in accordance with guidelines of the German potential analysis system (PotAS) for elite sports [31]. Of note, PotAS is a project of the Federal Ministry of the Interior, Building, and Community, Germany for evaluating performance elements of all sport disciplines according to specific criteria (organization, athlete development, sporting success) [31]. With respect to sporting success, the individual rank during the competition was evaluated using a graded point scale (i.e., rank 1 = 60 points, rank 2 = 40 points, rank 3 = 20 points, rank 4 = 10 points, rank 5 = 8 points, rank 6 = 6 points, rank 7 = 4 points, rank 8 = 2 points, attendance = 1 point, missing attendance = 0 points). The participants’ final points achieved were used for further analysis.

### 2.6. Statistical Analyses

All values are expressed as means and standard deviations (SD) after normal distribution of data was assessed and confirmed using the Shapiro–Wilk test. A 2 (time: pre, post) × 2 (sex: male, female) analysis of covariance with maturity level as a covariate was used to examine differences in anthropometry, body composition, and physical fitness over time in young male and female judo athletes. In the case of significant time × sex interactions, group-specific post-hoc analyses of variance on the factor time were calculated. Effect sizes were calculated by converting partial eta-squared to Cohen’s d. The magnitude of effect sizes was classified as small (0.2 ≤ *d* < 0.5), medium (0.5 ≤ *d* < 0.8), and large (*d* ≥ 0.8). [32] Additionally, a non-parametric rank correlation coefficient (Spearman’s Rho (ρ)) was calculated to assess relationships between changes in anthropometry, body composition, and physical fitness with success in competition. Correlation coefficients can be classified as small (ρ < 0.3), medium (0.3 ≤ ρ < 0.5), and large (ρ ≥ 0.5). Further, a stepwise linear regression (forward entry) was calculated with z-transformed sporting success scores as criterion variable and anthropometry, body composition, and physical fitness measures as potential predictor variables. One representative variable per test was used as predictor variable. Additionally, variables with correlation coefficients of *r* ≥ 0.8 were removed from the regression model to reduce multicollinearity. Predictor variables comprised body height, body mass, lean body mass, relative fat mass, CMJ height, RSI, and Y-balance test score of the dominant limb. The α level of significance was set a priori at *p* < 0.05. All analyses were performed using Statistical Package for Social Sciences (SPSS, IBM, Endicott, NY, USA) version 26.0.

## 3. Results

### 3.1. Training Data and Sporting Success

**Figure 1:**
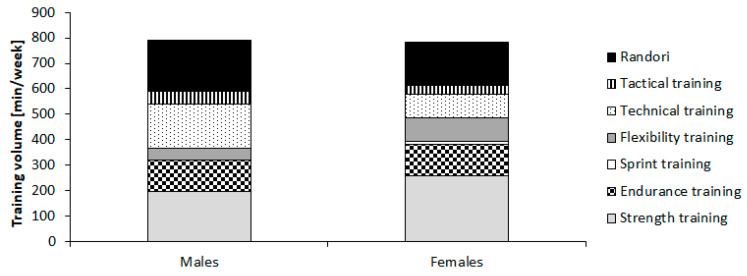
The distribution of the different training types across the entire season in young male and female judo athletes.

**Figure 2:**
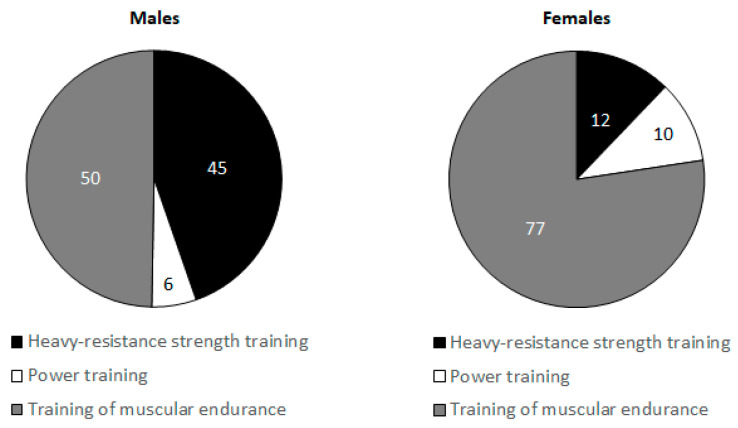
The distribution of the different strength training types across the entire season in young male and female judo athletes (in %).

Figure 1 illustrates the distribution of the different training types across the entire season for the examined cohort of young male and female judo athletes. Figure 2 shows the distribution of the different strength training types for young male and female judoka. In terms of sporting success, the achieved scores ranged from 0 to 60 points.

### 3.2. Anthropometry and Body Composition

**Figure 3:**
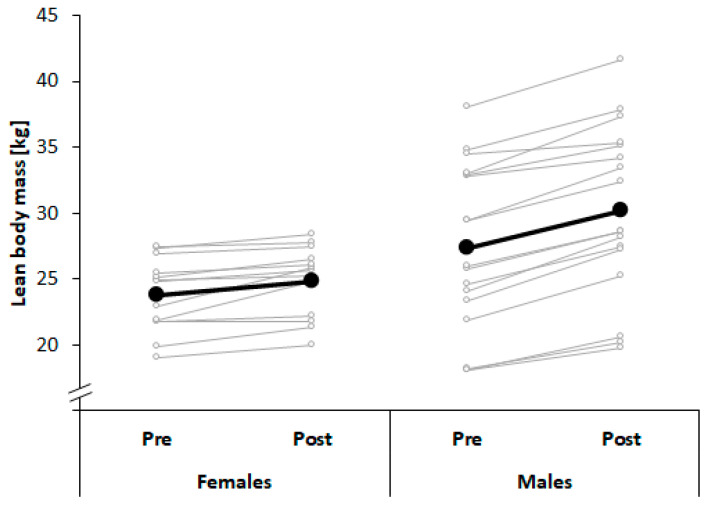
Changes in lean body mass across a period of 10 months of judo training in young male and female judo athletes.

Table 1 illustrates changes in anthropometry and body composition over the course of the entire season. Our findings showed significant and large-sized time × sex interactions for lean body mass (*p* < 0.05; *d* = 1.3). Post-hoc analysis showed significant and larger gains in lean body mass in young male (*p* < 0.001; *d* = 6.0) compared with female judo athletes (*p* < 0.01; *d* = 2.4; Figure 3). Further, significant large-sized time effects were observed for body height (*p* < 0.001; *d* = 4.3) and body mass (*p* < 0.001; *d* = 1.9), with higher values at post-test compared with pre-test, irrespective of factor sex. Additionally, significant large-sized effects of factor sex indicated larger body heights (*p* < 0.001; *d* = 1.8) and body masses (*p* < 0.001; *d* = 1.3) and lower body fat values (*p* < 0.05; *d* = 1.0) in males compared with females, irrespective of time.

### 3.3. Physical Fitness

**Figure 4:**
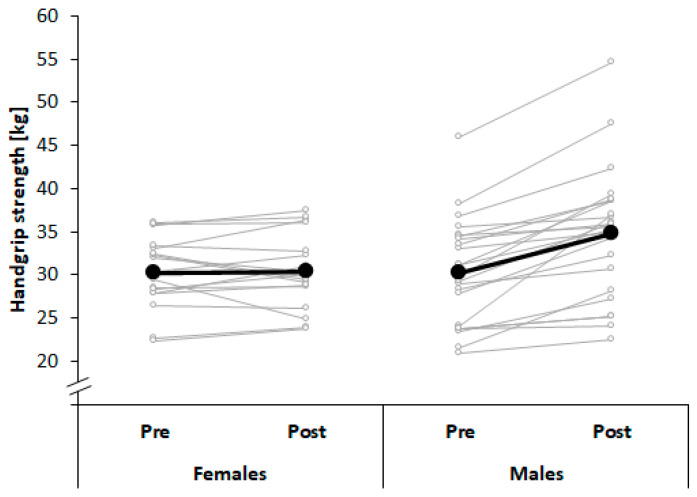
Changes in handgrip strength across a period of 10 months of judo training in young male and female judo athletes.

**Figure 5:**
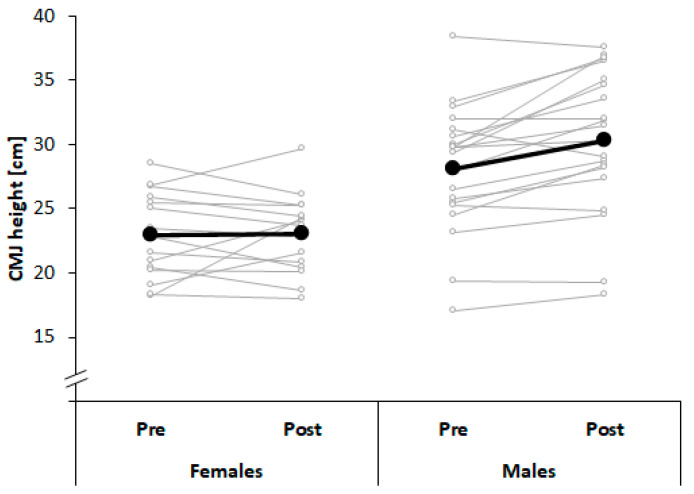
Changes in countermovement jump (CMJ) height across a period of 10 months of judo training in young male and female judo athletes.

Table 2 illustrates changes in components of physical fitness after a season of judo training. Results showed significant and medium-to-large-sized time × sex interaction effects for isometric handgrip strength and CMJ height (*p* < 0.05; 0.7 ≤ *d* ≤ 1.6). Post-hoc analyses revealed significantly increased handgrip strength and CMJ height in males (*p* < 0.01; 1.9 ≤ *d* ≤ 2.8), whereas no changes were observed in females (*p* > 0.05; Figure 4 and Figure 5). Further, significant and large-sized time effects were found for the Y-balance test scores of the dominant and non-dominant limb with larger scores at post-test compared with pre-test (*p* < 0.01; 1.2 ≤ *d* ≤ 1.3), irrespective of sex. Moreover, significant and large-sized effects of factor sex were associated with larger DJ height and RSI in males compared with females (*p* < 0.05; 0.9 ≤ *d* ≤ 1.1), irrespective of time.

### 3.4. Associations between Seasonal Changes in Anthropometry, Body Composition, and Physical Fitness with Sporting Success

Table 3 illustrates correlation coefficients for the associations between seasonal changes in anthropometry, body composition, and physical fitness and sporting success. Overall, our analysis revealed non-significant and small-to-moderate sized correlation coefficients (*p* > 0.05; −0.34 ≤ *ρ* ≤ 0.32). Additionally, there was no regression model which predicted sporting success.

## 4. Discussion

The purpose of this exploratory study was to describe and assess the effects of 10 months of regular judo training on anthropometry, body composition, and components of physical fitness and their associations with sporting success in young male and female judoka. The main findings can be summarized as followed: (i) young male judoka displayed larger gains in lean body mass, handgrip strength, and CMJ performance over the training period compared with female athletes; (ii) large-sized increases in body height and mass, as well as dynamic balance (Y-balance test), were shown from pre-to-post-test, irrespective of sex; (iii) body height/mass and DJ performance were higher while body fat mass was lower in young male compared with female judoka, irrespective of time; and (iv) seasonal changes in anthropometry, body composition, and physical fitness were not related to/did not predict sporting success in the main competition at the end of the season.

### 4.1. Anthropometry and Body Composition

Judo is a weight category sport, hence, body composition and in particular body fat and lean body mass are two important aspects that require systematic monitoring during the process of long-term athlete development [3,33]. In general, lower percentages of body fat mass and higher percentages of lean body mass increase relative strength levels in athletes [3]. Additionally, these two measures of body composition have been suggested to be relevant for success in the competition [34]. For instance, higher muscle and bone masses together with lower ectomorphy were associated with better performance in the Special Judo Fitness Test in athletes from the Spanish national judo team [34]. Generally, judo athletes seek to maximize the amount of lean body mass and minimize fat mass to decrease the overall body mass [3]. In an earlier study, Franchini et al. [35] compared elite (national and international medalists) versus sub-elite (non-medalists in national competitions) judo athletes regarding their limb circumferences used as an indicator of muscle mass. Authors reported higher muscle masses, particularly in the upper body, in elite compared with sub-elite judo athletes. In the same context, Drid et al. [6] compared male elite (internationally medalist) versus sub-elite (nationally medalist) judo athletes in terms of their anthropometric profile. Higher upper limb muscle masses were found in elite judo athletes compared with their sub-elite counterparts. In addition, lower percentages of body fat were recorded in international compared with national-level judo athletes [36,37]. Altogether, these findings indicate the importance of regularly monitoring measures of body composition such as body fat and lean body mass during athlete development of judoka.

Our analysis revealed significantly larger body fat masses in young female compared with male judoka. This is well in-line with previous studies [36,38]. For example, Little [36] revealed 10% higher body fat percentages in elite female judoka compared with their male peers. Further, Torres-Luque et al. [39] compared body composition parameters between male and female judoka aged 14 to 17 years. The authors reported higher percentages of body fat (24.6 vs. 12.7%) and less absolute lean body mass (41.6 vs. 52.7 kg) in females compared with males [39]. Generally, body fat percentage in female judoka aged 15 to 16 years ranged from 16 to 23% [40]. Likewise, in the present study relative body fat recorded during the pre- and post-tests in young female judo athletes ranged between 10.3 and 20.5%. Regarding body height and body mass, we found larger values in males compared with females, irrespective of time. This is in agreement with earlier studies [38,39] in young and adults judo athletes. These studies reported that male judoka were heavier and taller than females [38,39]. Moreover, our results indicated that young male judo athletes displayed larger gains in lean body mass over the training period compared with young female judoka. It has been demonstrated that body composition in males changes significantly throughout childhood and adolescence with increases in lean body mass and fat mass [41]. However, while the pattern of change in body composition is similar between males and females before puberty, it becomes more apparent around and after puberty [41]. Specifically, females gained higher fat mass and lower lean body mass compared with males after puberty [41]. These differences are particularly attributed to the higher concentration of circulating androgens (e.g., testosterone) in males compared with females [41,42]. The increase in circulating androgens in males results in a rapid increase in lean body mass, a small increase in fat mass, and an overall reduction in the percentage of body fat [41,42]. Thus, hormonal levels may explain the larger gains in lean body mass across time in young male versus female judo athletes observed in the present study. In summary, sex-specific differences in anthropometry and body composition were observed in young judoka which could be substantiated across 10 months of judo training and/or growth/maturation.

### 4.2. Measures of Physical Fitness

Muscle strength is a fundamental component of physical fitness in many sports that needs to be developed at an early age to achieve high competitive performance on the elite level [9]. In this regard, muscle strength and power were identified as decisive physical fitness attributes in combat sports such as judo [3]. For instance, given that judoka have to grip the opponent’s suit to prepare for an attack, high isometric grip strength performance is crucial for success in the sport [3]. In terms of muscle power, Fagerlund [43] showed that elite judoka outperformed their recreational counterparts. Similarly, Drid et al. [6] compared between male elite vs. sub-elite judo athletes regarding their lower limb muscle power. They revealed higher performance in the maximal power test (i.e., 8 secs all-out cycling on a cycle ergometer) in the elite compared with the sub-elite group. In this regard, the present study showed significant increases in handgrip strength throughout the season in young male but not in female judo athletes. Additionally, our results showed significant improvements in CMJ height throughout the training season only in males. Previously, Franchini et al. [44] compared isometric handgrip strength between male and female judoka and revealed higher strength values in males. Further, in a sample of Spanish adolescent elite judoka, Torres-Luque et al. [39] observed higher isometric handgrip strength in males compared with females, whereas CMJ heights were not significantly different. Of note, the isometric handgrip strength values and CMJ performance observed in the study of Torre-Luque et al. [39] were similar to the strength levels in German young judoka in our study (26.6 to 37.7 kg vs. 27.2 to 37.2 kg, and 27.7 to 30.8 cm vs. 21.7 to 31.2 cm, respectively). Nevertheless, to the best of our knowledge, the present study is the first to examine the long-term development of muscle strength and power in young judoka across a 10-month training period. Interestingly, the finding of superior strength/power gains in males compared with females comply with our findings in body composition (i.e., lean body mass). Of note, lean body mass is determinant for muscle strength and power in combat athletes [3,45]. Therefore, it seems reasonable to assume that the larger, training- and/or growth/maturation-induced increases in lean body mass partly contributed to the larger gains in strength/power performance in young male versus female judo athletes. In this context, it has previously been shown that female jump distances are about 5–10% lower than males’ before puberty (e.g., nine years) accentuating to >15% after puberty (e.g., 14 to 15 years) [46]. Interestingly, in our study strength training amounted to 25% of the total training volume in females compared with 20% in males. In fact, in terms of the type of strength training, females performed less high-resistance strength exercises (12% vs. 45%) and more muscular endurance exercises (77% vs. 50%) than males over the season. Accordingly, the non-significant increase in measures of maximal strength and power in females may reflect an inadequacy of the training load to stimulate these important fitness qualities.

In terms of balance performance, the present study showed significant and large-sized increases in dynamic balance (i.e., Y-balance test) over the training period in young male and female judo athletes. The ability to maintain balance should be mastered during the early stages of long-term athlete development [9,47]. In fact, gaining proficiency in postural control at an early age could contribute to increase the levels of other fitness components and/or the efficiency of executing more sophisticated movement skills at later stages of long-term athlete development [47,48]. Additionally, improving postural control may also mitigate the likelihood of a child sustaining an injury during the performance of complex movement skills or during sports practice [49,50]. Interestingly, Perrin et al. [51] demonstrated better balance performance (i.e., single-leg stance test) in adult judo athletes compared with non-athlete controls and partly even compared with ballet dancers (i.e., eyes-closed single-leg stance test; mean age: 20 to 35 years). Further and in agreement with our findings, Walaszek et al. [52] demonstrated that six months of regular judo training compared with a passive control period resulted in significant improvements in single-leg stance performance in young male children aged six years. Therefore, regular judo training and/or growth/maturation may be effective to improve (steady-state) balance performance in young athletes.

### 4.3. Associations of Seasonal Changes in Anthropometry, Body Composition, and Physical Fitness with Sporting Success

Our findings revealed that changes in anthropometry, body composition, and physical fitness were not associated with sporting success in competition. These results indicate that young judoka with larger seasonal gains in body height, lean body mass, or maximal strength are not necessarily those athletes with more successful performance in competition. This was also reinforced by findings from our regression analysis. Of note, judo has previously been classified as a late specialization sport [15]. More precisely, judo requires specialization at approximately age 15 to 16 years for males and 13 to 14 years for females [15]. Irrespective of the sport, a premise of late specialization (or early diversification) is to train and test a broad foundation of physical fitness and diverse motor skills during the early stages of long-term athlete development before developing sport-specific performance [15,53,54]. Consequently, judo training programs during the early stages of long-term athlete development are not supposed to target sporting success at young ages but rather at the elite level. In fact, across a 10-year observation period, only 7% and 5% of male and female judo medalists (state-level competition, 9–20+ years old), respectively, maintained their competition levels. [55] Thus, the late specialization approach may partly explain the lack of associations between changes in anthropometry, body composition, and physical fitness with sporting success in judo competition at a relatively young age. Further, other factors than anthropometry, body composition, and physical fitness are considered important for judo performance, such as technique, tactical–cognitive skills, and visual tracking [15,56]. Future studies should examine the role of these factors for performance during competition particularly in young judo athletes. Additionally, our correlation coefficients need to be interpreted with caution because ties were observed particularly at the lower limits of the value range [57].

### 4.4. Limitations

The present study has some limitations that need to be discussed. First, the lack of a passive control group should be acknowledged as a methodological limitation of this study. However, the inclusion of a passive control group (i.e., no judo training) is impossible in a sub-elite athletic setting because we cannot expect athletes to stop training for an entire season. Second, the maturity-related differences in performance are a confounding factor that could have affected variations in anthropometry, body composition, and fitness components in our sample. However, the applied statistical approach of an analysis of covariance allowed us to control for differences in maturity level. Third, the testing battery did not comprise sport-specific performance tests such as the Special Judo Fitness Test [2] or assessment of judo-specific pulling kinetics using the JERGo© system [58]. This needs to be considered in future research. Moreover, the training data was recorded on the group level allowing for descriptive analysis only. As such, the lack of training monitoring on an individual basis constitutes another limitation of this study. Further, no detailed information about the global physical activity, content of each training type, diet, and/or injury prevalence were provided in this study. This should be considered in future studies. Finally, the lack of significant strength and power changes in female athletes could (partly) be justified by our methodological testing (i.e., two testing points one at the beginning and one at the end of the training season). Future studies might include additional testing points (e.g., after single training blocks/cycles) to provide more detailed information on the temporal changes in anthropometry, body composition, and physical fitness across the season in young judo athletes.

## 5. Conclusions

The present exploratory study revealed that young male judoka displayed larger gains in lean body mass, handgrip strength, and CMJ performance over a 10-month training period compared with female athletes. It can be argued that the increments in lean body mass partly contributed to the gains in strength/power performance predominantly in young male judo athletes. Additionally, regular judo training and/or growth/maturation appeared to be an effective means to improve (steady-state) balance performance in young male as well as female judoka. Sex-specific differences in anthropometry (i.e., body height/mass) and body composition (i.e., body fat mass) were found in young judo athletes that could have been attributed to differences in hormonal status. A lack of associations between seasonal changes in anthropometry, body composition, and physical fitness with sporting success in competition may be attributed to the late specialization approach in judo. Young judo athletes are advised to conduct judo training on a regular basis to potentially contribute to gains in anthropometry, body composition, and physical fitness. In particular, young male judoka appear to benefit from judo training in terms of body composition (i.e., lean body mass) and physical fitness (i.e., muscle strength, muscle power). Therefore, more adapted long-term judo training programs for young females are needed in the future to enhance the chances for continuous improvements in important fitness measures such as muscle strength and power during the course of long-term athlete development.

## Figures and Tables

**Table 1:** Changes in anthropometry and body composition in young male and female judo athletes.

Parameter	Unit	Females						Males						Statistics		
			Pre		Post		∆ (%)		Pre		Post		∆ (%)	Time	Sex	Time × Sex
		*n*	M	SD	M	SD		*n*	M	SD	M	SD		*p* (*d*)	*p* (*d*)	*p* (*d*)
Body height	cm	18	158.7	5.2	161.7	5.5	1.9	23	168.7	5.0	171.6	4.7	1.7	<0.001 (4.34)	<0.001 (1.77)	0.787 (0.09)
Body mass	kg	13	47.4	9.8	50.6	11.2	6.8	18	60.9	9.5	65.3	9.1	7.2	<0.001 (1.90)	0.002 (1.30)	0.420 (0.31)
Body mass index	kg/m^2^	12	19.3	3.4	19.7	3.5	1.9	18	21.5	3.2	22.3	2.7	3.8	0.081 (0.70)	0.094 (0.67)	0.346 (0.37)
Lean body mass	kg	13	19.8	2.8	21.0	3.3	6.2	18	30.2	2.7	32.9	2.7	8.9	<0.001 (3.18)	<0.001 (3.50)	0.002 (1.26)
Body fat	%	13	19.3	8.1	20.5	8.3	5.9	18	10.7	7.8	10.1	6.8	−5.3	0.905 (0.06)	0.006 (1.13)	0.131 (0.59)

Post-test means (M) and standard deviations (SD) are adjusted for maturity level; d = effect size (Cohen’s d), *n* = sample size, *p* = alpha error, ∆ = relative change.

**Table 2:** Changes in physical fitness measures in young male and female judo athletes.

Parameter	Females						Males						Statistics		
		Pre		Post		∆ (%)		Pre		Post		∆ (%)	Time	Sex	Time × Sex
	*n*	M	SD	M	SD		*n*	M	SD	M	SD		*p* (*d*)	*p* (*d*)	*p* (*d*)
Handgrip strength (kg)	17	27.8	5.8	27.2	6.6	−2.1	23	32.0	5.5	37.2	6.4	16.2	0.028 (0.75)	0.002 (1.10)	<0.001 (1.58)
CMJ height (cm)	15	21.7	4.7	21.7	5.1	0.2	20	29.0	4.6	31.2	5.0	7.6	0.104 (0.59)	<0.001 (1.70)	0.044 (0.74)
DJ height (cm)	15	19.5	4.9	18.4	5.5	−5.8	20	24.7	4.8	24.4	5.4	−1.4	0.106 (0.59)	0.005 (1.07)	0.490 (0.25)
Reactive strength index (m/s)	15	1.0	0.3	0.9	0.3	−1.9	20	1.2	0.3	1.2	0.3	0.3	0.686 (0.14)	0.019 (0.87)	0.763 (0.11)
Y-balance test score (dom.) (%)	14	104.6	7.8	111.5	10.2	6.6	20	105.3	7.5	110.9	9.9	5.2	0.001 (1.35)	0.991 (0)	0.730 (0.13)
Y-balance test score (non-dom.) (%)	14	106.0	8.5	111.3	9.0	5.0	21	104.7	8.2	110.8	8.7	5.9	0.002 (1.18)	0.748 (0.11)	0.823 (0.09)

Post-test means (M) and standard deviations (SD) are adjusted for maturity level; CMJ = countermovement jump, *d* = effect size (Cohen’s d), DJ = drop jump, *n* = sample size, *p* = alpha error, ∆ = relative change.

**Table 3:** Correlation between seasonal changes in anthropometry/body composition and physical fitness, and success in main competition in young male and female judo athletes.

Seasonal Changes in (…)	Competition
***Anthropometry/body composition***	
Body height	−0.17
Body mass	−0.34
Body mass index	−0.33
Lean body mass	−0.11
Body fat	−0.07
***Physical fitness***	
Handgrip strength	0
CMJ height	−0.25
DJ height	0.06
Reactive strength index	0.32
Y-balance test score (dom.)	−0.25
Y-balance test score (non-dom.)	−0.19

Numbers indicate non-parametric correlation coefficient Spearman’s *Rho*; CMJ = countermovement jump, DJ = drop jump.

## References

[1] Akoto R., Lambert C., Balke M., Bouillon B., Frosch K.H., Hoher J. (2018). Epidemiology of injuries in judo: A cross-sectional survey of severe injuries based on time loss and reduction in sporting level. Br. J. Sports Med..

[2] Chaabene H., Negra Y., Bouguezzi R., Capranica L., Franchini E., Prieske O., Hbacha H., Granacher U. (2018). Tests for the Assessment of Sport-Specific Performance in Olympic Combat Sports: A Systematic Review With Practical Recommendations. Front. Physiol..

[3] Franchini E., Del Vecchio F.B., Matsushigue K.A., Artioli G.G. (2011). Physiological profiles of elite judo athletes. Sports Med. (Auckl. N. Z.).

[4] Detailed Explanation of the IJF Judo Refereeing Rules Effective from 01 January 2018. https://www.ijf.org/news/show/detailed-explanation-of-the-ijf-judo-refereeing-rules.

[5] Franchini E., Brito C.J., Fukuda D.H., Artioli G.G. (2014). The physiology of judo-specific training modalities. J. Strength Cond. Res..

[6] Drid P., Casals C., Mekic A., Radjo I., Stojanovic M., Ostojic S.M. (2015). Fitness and anthropometric profiles of international vs. national judo medalists in half-heavyweight category. J. Strength Cond. Res..

[7] Kim J., Cho H.C., Jung H.S., Yoon J.D. (2011). Influence of performance level on anaerobic power and body composition in elite male judoists. J. Strength Cond. Res..

[8] Bourdon P.C., Cardinale M., Murray A., Gastin P., Kellmann M., Varley M.C., Gabbett T.J., Coutts A.J., Burgess D.J., Gregson W. (2017). Monitoring Athlete Training Loads: Consensus Statement. Int. J. Sports Physiol. Perform..

[9] Lloyd R.S., Oliver J.L., Faigenbaum A.D., Howard R., De Ste Croix M.B., Williams C.A., Best T.M., Alvar B.A., Micheli L.J., Thomas D.P. (2015). Long-term athletic development-part 1: A pathway for all youth. J. Strength Cond. Res..

[10] Detanico D., Kons R.L., Fukuda D.H., Teixeira A.S. (2020). Physical Performance in Young Judo Athletes: Influence of Somatic Maturation, Growth and Training Experience. Res. Q. Exerc. Sport.

[11] Ullrich B., Pelzer T., Oliveira S., Pfeiffer M. (2016). Neuromuscular Responses to Short-Term Resistance Training With Traditional and Daily Undulating Periodization in Adolescent Elite Judoka. J. Strength Cond. Res..

[12] Franchini E., Branco B.M., Agostinho M.F., Calmet M., Candau R. (2015). Influence of linear and undulating strength periodization on physical fitness, physiological, and performance responses to simulated judo matches. J. Strength Cond. Res..

[13] Zauner C.W., Maksud M.G., Melichna J. (1989). Physiological considerations in training young athletes. Sports Med. (Auckl. N.Z.).

[14] McManus A.M., Armstrong N. (2011). Physiology of elite young female athletes. Med. Sport Sci..

[15] Balyi I., Way R., Higgs C. (2013). Long-Term Athlete Development.

[16] Wind A.E., Takken T., Helders P.J.M., Engelbert R.H.H. (2010). Is grip strength a predictor for total muscle strength in healthy children, adolescents, and young adults?. Eur. J. Pediatrics.

[17] Markovic G., Dizdar D., Jukic I., Cardinale M. (2004). Reliability and factorial validity of squat and countermovement jump tests. J. Strength Cond. Res..

[18] Schwiertz G., Brueckner D., Schedler S., Kiss R., Muehlbauer T. (2019). Performance and reliability of the Lower Quarter Y Balance Test in healthy adolescents from grade 6 to 11. Gait. Posture.

[19] Mirwald R.L., Baxter-Jones A.D., Bailey D.A., Beunen G.P. (2002). An assessment of maturity from anthropometric measurements. Med. Sci. Sports Exerc..

[20] Chaabene H., Negra Y., Bouguezzi R., Mkaouer B., Franchini E., Julio U., Hachana Y. (2017). Physical and Physiological Attributes of Wrestlers: An Update. J. Strength Cond. Res..

[21] McLester C.N., Nickerson B.S., Kliszczewicz B.M., McLester J.R. (2018). Reliability and Agreement of Various InBody Body Composition Analyzers as Compared to Dual-Energy X-ray Absorptiometry in Healthy Men and Women. J. Clin. Densitom. Off. J. Int. Soc. Clin. Densitom..

[22] Gerodimos V. (2012). Reliability of handgrip strength test in basketball players. J. Hum. Kinet..

[23] Coren S. (1993). The lateral preference inventory for measurement of handedness, footedness, eyedness, and earedness: Norms for young adults. Bull. Psychon. Soc..

[24] Glatthorn J.F., Gouge S., Nussbaumer S., Stauffacher S., Impellizzeri F.M., Maffiuletti N.A. (2011). Validity and reliability of Optojump photoelectric cells for estimating vertical jump height. J. Strength Cond. Res..

[25] Kibele A. (1999). Technical note. Possible errors in the comparative evaluation of drop jumps from different heights. Ergonomics.

[26] Prieske O., Muehlbauer T., Krueger T., Kibele A., Behm D., Granacher U. (2015). Sex-specific effects of surface instability on drop jump and landing biomechanics. Int. J. Sports Med..

[27] Filipa A., Byrnes R., Paterno M.V., Myer G.D., Hewett T.E. (2010). Neuromuscular training improves performance on the star excursion balance test in young female athletes. J. Orthop. Sports Phys. Ther..

[28] Plisky P.J., Rauh M.J., Kaminski T.W., Underwood F.B. (2006). Star Excursion Balance Test as a predictor of lower extremity injury in high school basketball players. J. Orthop. Sports Phys. Ther..

[29] Sipe C.L., Ramey K.D., Plisky P.P., Taylor J.D. (2019). Y-Balance Test: A Valid and Reliable Assessment in Older Adults. J. Aging and Phys. Act..

[30] Bastien M., Moffet H., Bouyer L., Perron M., Hébert L.J., Leblond J. (2014). Concurrent and discriminant validity of the Star Excursion Balance Test for military personnel with lateral ankle sprain. J. Sport Rehabil..

[31] Büsch D.H., Rebel M., Wendt R., Horn A., Granacher U. (2018). One year PotAS Commission- Objectives, tasks, and current state. Leistungssport.

[32] Cohen J. (1988). Statistical Power Analysis for the Behavioral Sciences.

[33] Torres-Luque G., Hernández-García R., Escobar-Molina R., Garatachea N., Nikolaidis P.T. (2016). Physical and Physiological Characteristics of Judo Athletes: An Update. Sports.

[34] Casals C., Huertas J.R., Franchini E., Sterkowicz-Przybycień K., Sterkowicz S., Gutiérrez-García C., Escobar-Molina R. (2017). Special Judo Fitness Test Level and Anthropometric Profile of Elite Spanish Judo Athletes. J. Strength Cond. Res..

[35] Franchini E., Takito M., Kiss M., Strerkowicz S. (2005). Physical fitness and anthropometrical differences between elite and non-elite judo players. Biol. Sport.

[36] Little N. (1991). Physical performance attributes of junior and senior women, juvenile, junior, and senior men judokas. J. Sports Med. Phys. Fit..

[37] Fukuda D.H., Stout J.R., Kendall K.L., Smith A.E., Wray M.E., Hetrick R.P. (2013). The effects of tournament preparation on anthropometric and sport-specific performance measures in youth judo athletes. J. Strength Cond. Res..

[38] Franchini E., Rodríguez Huertas J., Sterkowicz S., Carratalá V., Gutiérrez-García C., Escobar-Molina R. (2011). Anthropometrical profile of elite Spanish Judoka: Comparative analysis among ages. Arch. Budo.

[39] Torres-Luque G., Hernandez-Garcia R., Garatachea N., Nikolaidis P. (2015). Anthropometric characteristics and neuromuscular function in young judo athletes by sex, age and weight category. Sport Sci. Health.

[40] Boisseau N., Vera-Perez S., Poortmans J. (2005). Food and fluid intake in adolescent female judo athletes before competition. Pediatric Exerc. Sci..

[41] Beunen G., Malina R.M., Hebestreit H., Bar-Or O. (2008). Growth and biologic maturation: Relevance to athletic performance. The Young Athlete.

[42] Malina R.M., Bouchard C., Bar-Or O. (2004). Growth, Maturation, and Physical Activity.

[43] Fagerlund R. (1991). Strength profile of Finnish judoists-measurement and evaluation. Biol. Sport.

[44] Franchini E., Takito M., Matheus L., Vieira D.B., Kiss M.A.P. (1997). Composição corporal, somatotipo e força isométrica em atletas da seleção brasileira universitária de judô. Ambito Med. Esportiva.

[45] Kavvoura A., Zaras N., Stasinaki A.-N., Arnaoutis G., Methenitis S., Terzis G. (2018). The Importance of Lean Body Mass for the Rate of Force Development in Taekwondo Athletes and Track and Field Throwers. J. Funct. Morphol. Kinesiol..

[46] Catley M.J., Tomkinson G.R. (2013). Normative health-related fitness values for children: Analysis of 85347 test results on 9–17-year-old Australians since 1985. Br. J. Sports Med..

[47] Gebel A., Prieske O., Behm D.G., Granacher U. (2020). Effects of Balance Training on Physical Fitness in Youth and Young Athletes: A Narrative Review. Strength Cond. J..

[48] Granacher U., Lesinski M., Busch D., Muehlbauer T., Prieske O., Puta C., Gollhofer A., Behm D.G. (2016). Effects of Resistance Training in Youth Athletes on Muscular Fitness and Athletic Performance: A Conceptual Model for Long-Term Athlete Development. Front. Physiol..

[49] Mickle K.J., Munro B.J., Steele J.R. (2011). Gender and age affect balance performance in primary school-aged children. J. Sci. Med. Sport.

[50] Willems T.M., Witvrouw E., Delbaere K., Mahieu N., De Bourdeaudhuij L., De Clercq D. (2005). Intrinsic risk factors for inversion ankle sprains in male subjects: A prospective study. Am. J. Sports Med..

[51] Perrin P., Deviterne D., Hugel F., Perrot C. (2002). Judo, better than dance, develops sensorimotor adaptabilities involved in balance control. Gait Posture.

[52] Walaszek R., Sterkowicz S., Chwała W., Sterkowicz-Przybycień K., Walaszek K., Burdacki M., Kłys A. (2017). Assessment of the impact of regular judo practice on body posture, balance, and lower limbs mechanical output in six-year-old boys. J. Sports Med. Phys. Fit..

[53] Côté J., Baker J., Abernethy B. (2007). Practice and play in the development of sport expertise. Handbook of Sport Psychology.

[54] Myer G.D., Jayanthi N., DiFiori J.P., Faigenbaum A.D., Kiefer A.W., Logerstedt D., Micheli L.J. (2016). Sports Specialization, Part II: Alternative Solutions to Early Sport Specialization in Youth Athletes. Sports Health.

[55] Julio U.F., Takito M.Y., Mazzei L., Miarka B., Sterkowicz S., Franchini E. (2011). Tracking 10-Year Competitive Winning Performance of Judo Athletes across Age Groups. Percept. Mot. Ski..

[56] Miarka B., Cury R., Julianetti R., Battazza R., Julio U.F., Calmet M., Franchini E. (2014). A comparison of time-motion and technical-tactical variables between age groups of female judo matches. J. Sports Sci..

[57] Liu Y. A Short Note on Spearman Correlation: Impact of Tied Observations. https://papers.ssrn.com/sol3/papers.cfm?abstract_id=2933193.

[58] Helm N., Prieske O., Muehlbauer T., Kruger T., Chaabene H., Granacher U. (2018). Validation of a New Judo-Specific Ergometer System in Male Elite and Sub-Elite Athletes. J. Sports Sci. Med..

